# The Foreign Journals: Pathology, Practical Medicine, and Therapeutics

**Published:** 1842-07

**Authors:** 


### PATHOLOGY, PRACTICAL MEDICINE, AND THERAPEUTICS.

### *On the Typhus which prevailed at Rheims in* 1833 *and* 1840. By M. Landouzy, Professor at the Medical School at Rheims.

The object of M. Landouzy is to examine into the analogies between the typhoid fever of France and the typhus which prevails in England.

His observations were made in the prison at Rheims, where fever broke out on October 1st, 1839, and continued to prevail until June in the following year, during which time 138 persons were attacked by the disease, of whom 17 died. Of these persons 103 were prisoners, 35 were physicians or attendants on the sick, of whom 9 died; whence it appears, that the disease proved more fatal to those who, coming from without, contracted it by contagion than to such as were seized directly by it in prison. It is remarkable that the epidemic was entirely confined to the prisoners on the untried side, while the convicted criminals entirely escaped. The prison which is constructed to hold from 80 to 100, usually contains 130 or 140, and in August and September, 1839, there were in it as many as 180 prisoners. The unusual crowding, too, during the autumn months was entirely confined to the untried prisoners, to whom likewise the fever was limited.

The symptoms, which are carefully detailed, do not differ from those observed in the recent epidemic in London. Nine tenths of the patients presented some degree of stupor; perfect coma, however, existed only in one case out of twelve. Delirium occurred in every case, but it was of a noisy character only in one case out of ten, and M. Landouzy thinks that it is always milder and less boisterous than in the *fièvre typhöide.* A petechial eruption was observed in every case but one. It usually appeared from the fourth to the fifth day, and disappeared from the tenth to the eighteenth. These spots did not disappear under pressure, and were abundant and confluent in proportion to the severity of the disease. Besides the petechiæ, lenticular spots of a rosy red, the spots of typhoid fever, existed in many instances. This rosy exanthema was always incomparably less abundant than the petechial eruption and was exclusively confined to the chest or the upper third of the abdomen, while the latter extended over nearly the whole body. They differed likewise in the date of their appearance, the petechiæ often showing themselves from the very onset of the disease, while the red spots were never visible before the tenth or twelfth day. Diarrhœa was present only in four cases, but sibilus was heard throughout the lungs in almost every instance.

The contagious nature of the disease was placed beyond all doubt by the circumstance that 35 of the attendants were attacked by the disease.

Of the 17 patients who died, the bodies of 6 only were examined; but in all the lesions found in the intestines were precisely those which are characteristic of the *fièvre typhöide.* Nothing particular was observed in any of the other organs, nor were any of those superficial erosions of the brain noticed which M. Piednagel and M. Louis mention, and which the former says are always met with when well marked cerebral symptoms have existed during the course of the fever.

The writer next examines the question of the identity or non-identity of the typhus of Rheims with the typhoid fever, and concludes—

That if, in all future epidemics of camp, jail, or hospital typhus, there should be found, as in the fever at Rheims, a complete absence of lesion of the spleen, as well as great difference among the functional disturbances which constitute the symptomatological character of typhoid fever, we should not be warranted in regarding typhus and dothenteritis more than as analogous diseases. If, on the other hand, different symptoms should be wanting in different epidemics, the conclusion would be warranted, that the two diseases are identical, and that their differences are owing to peculiarities in the epidemic constitution under which they arose.

In the present state of the question, M. Landouzy concludes that the jail typhus of Rheims and the typhoid fever present too close a resemblance not to be regarded as analogous, while their differences are too numerous to warrant the opinion of their being identical.

*Archives Générales de Médecine. Jan. et Mars,* 1842.

---

*Statistical Researches into the Etiology of Pulmonary Phthisis.*
By Dr. BRIQUET, of the Hôpital Cochin.

THIS paper is founded on an investigation into various particulars connected with the history of 109 phthisical patients in whom the disease was far advanced, and likewise on data furnished by all the deaths from phthisis in the hospital between January 1st, 1838, and January 1st, 1841, being 182 in number.

The conclusions (for which only we have space,) at which M. Briquet arrives are:

1. That during the past three years one tenth more of men than of women have been received into the Hôpital Cochin affected with phthisis: a result directly contrary to those obtained by MM. Lombard and Louis.

2. In at least a third of the patients phthisis was distinctly hereditary, and predisposition to the disease seemed more frequently to come from the father than the mother.

3. No immunity from the disease is afforded by the circumstance of being born of parents who are natives of the country, or by being brought up in the country.

4. Tall stature, a slender frame, an ill formed chest, and convexity from the root to the point of the nails are the only external characteristics of phthisical diathesis.

5. It occurred very seldom that the circumference of the upper part of the chest was less than that of the lower part: a fact directly contrary to the assertion of M. Hertz.

6. Those callings in the pursuit of which there is discomfort, want of exercise and of pure air present a greater number of phthisical persons than is to be found among those who pursue different occupations.

7. A third of these patients were more subject to catarrh than other persons, and were more sensible of cold.

8. In three fifths of the patients phthisis developed itself between twenty and thirty years of age, but more than two thirds of those whose parents had suffered from consumption became phthisical before their thirtieth year; while, of those whose parents had not been healthy, half did not show symptoms of phthisis till after thirty.

9. In four fifths of the patients there existed predisposition to phthisis, and in three fifths this predisposition was acquired.

10. Cold is the most powerful cause of the acquired predisposition; next to which are misery, privation, and distress of mind.

11. Phthisis is most frequent in cold seasons, and when there are many variations in the atmosphere.

12. Four tenths of the patients had not been exposed to the influence of any occasional cause of phthisis, but in most there existed a strong predisposition to the disease.

Five tenths had been exposed to and suffered greatly from some exciting cause, and this cause was in almost every instance cold and damp.

*Revue Médicale. Feb.* 1842.

---

*On a peculiar Alteration of the Cerebral Substance.*
By M. MAX. DURAND FARDEL.

M. FARDEL has occasionally observed in the white substance of the hemispheres of the brain vascular canals, which present, on a section being made, an appearance precisely similar to that with which anatomists are familiar at those parts which are called the perforated plates of the brain. This condition M. Fardel regards as the result of a pathological process, and purposes to distinguish it by the name of "sieve-like condition of the brain,"—*état criblé du cerveau.*

These little holes are usually surrounded by perfectly healthy cerebral substance; they are circular, with well-defined edges scattered irregularly through the brain, varying in diameter, and unchanged in form by a stream of water. If water is allowed to flow on them for any time the cerebral substance is gradually washed away, and the little holes are seen to have been the artificial openings of canals, each of which contained a vessel.

The alteration is regarded by M. Fardel as the result of a general dilatation of the vessels of the brain produced by frequent congestion. Dilatation of the blood-vessels is by no means a rare appearance in the brains of old persons. It is in general especially-well marked in the corpora striata, where small canals are frequently seen, each of which contains a vessel that, being empty of blood, appears disproportionately small in comparison with the canal wherein it runs. This state is not in general associated with any alteration of the cerebral substance, other than an appearance of dilatation of the vessels of the hemispheres: congestion of the brain, of necessity, causes a temporary dilatation of the vessels of the organ; its frequent recurrence must compress the brain around each vessel and form in its substance those canals which are so evident when the vessels are empty and collapsed after death. The sieve-like condition of the brain is sometimes found unassociated with any other lesion; at other times, however, it coexists with the various forms of softening of the brain, and especially with that general *ramollissement* of the cortical layer of the convolutions which M. Calmeil has described as peculiar to the general paralysis of the insane, with induration of the brain, &c. Evidence exists to prove that the connexion between the *état criblé* of the brain and other lesions with which it is associated is not merely accidental; thus, in one instance in which idiocy, attended with occasional attacks of mania, followed a blow on the head, induration of the brain and the *sieve-like condition* were found confined to that hemisphere of the cerebrum on which the injury had been inflicted. It is not unusual in cases of chronic softening of the brain to meet with this sieve-like appearance for some distance around the softened part, betokening the previous existence of dilatation of the vessels. In the same brain, too, M. Fardel has met with recent *ramollissement* and injection of the vessels and old *ramollissement* with the *état criblé.*

From his observations, many of which are detailed at length, the writer concludes that,

The *état criblé* of the brain is produced by the presence of a great number of small canals, perforated in the cerebral tissue, each containing a little vessel, to the dilatation of which their formation is doubtless owing. These canals, the existence of which is normal in some parts of the periphery of the brain, appear in a rudimentary state without being necessarily morbid in some persons in advanced life.

Their usual seat is in the cerebral hemispheres, especially beneath the convolutions; but they are likewise met with in the cerebral protuberance, and in the medulla oblongata.

Though somewhat different in appearance, the little cavities so often seen in the corpora striata are probably of a similar nature.

The general or partial dilatation of a great number of the vessels of the brain appears to be owing to chronic sanguineous congestion, or to the frequent recurrence of congestion of the organ.

This opinion seems sufficiently warranted by the phenomena observed during life, as well as by the alterations which are found after death to coexist with the *état criblé* of the brain.

Twice this condition existed uncombined with any other appreciable lesion of the brain. In one of these cases there was simple dementia, in the other, dementia with general paralysis.

It is found associated with superficial *ramollissement* of the convolutions in insane persons affected with general paralysis, with general or partial induration of the brain, and in the centre of or around portions of softened brain.

This state probably existed but escaped notice in cases where grave cerebral symptoms were not found to have given rise to any lesion appreciable after death.

*Gazette Médicale. Jan.* 8, 15, 1842.

---

*Peculiar Matter secreted on the Surface of the Hands of a Gouty Person after severe attacks of Gout.* By Dr. PETIT.

THE patient who furnished this secretion was fifty-six years old, of a strong constitution, and addicted to good living. He had been subject to gout ever since his twenty-fourth year, and during the attacks of gout his urine frequently deposited a red sediment. After severe attacks he had observed a tenacious white matter form on his hands. Four grains and half of this substance, examined under the microscope, presented a number of transparent crystals. By chemical analysis it was ascertained to be composed of albumen in large quantity—about four fifths, of lactic and phosphoric acid, chloride of soda and phosphate of lime, and evident traces of urate of soda.

*Gazette des Hôpitaux. Feb.* 22, 1842. (*From the Journal de Pharmacie.*)

---

*Singular case of Suppuration of the Arachnoid.* By Dr. WAGNER, of Vienna.

AN infantry soldier in the Austrian service, of excellent conduct, and who had been the subject of no disease during the seven years of his military life, complained, on the 17th of November, 1839, of an affection resembling gastritis, for which he took on the following day a saline mixture in large doses, and derived some benefit therefrom; yet betook himself to bed at mid-day, (November 18,) and after sleeping for one hour, awoke with signs of disturbed intelligence, which gradually passed into profound unconsciousness, accompanied with strong convulsions. General and local bloodletting was fruitlessly had recourse to, the patient dying at half-past four in the morning of the 19th.

The principal morbid appearances internally were as follows: The dura mater had a parchment feel, and was not much injected; in the sinus of the falciform process there was a small quantity of blood: after removal of the dura mater, the whole surface of the cerebrum was seen to be covered with a yellow, thin pus, of such a consistence that it fell in drops from the brain, and could be easily wiped off with the back of the scalpel; there was slight depression of the brain over the ophthalmic ventricle; the arachnoid membrane was so entirely disorganized that no trace of it could be detected; the vessels of the pia mater were distended with blood, even in their minutest ramifications, and between the cerebral convolutions there was no inconsiderable quantity of pus; the cerebral substance was pappy, and studded with bloody points; the lateral ventricles were empty, but their walls completely softened; the whole lower surface of the cerebrum and the whole cerebellum were without a trace of arachnoid membrane, but covered with pus and much softened; the air-passages were slightly reddened; the *blood-vessels of the throat were greatly distended.* It may be added generally, that there were signs of disease in all or most of the other organs of the body, as the lungs, heart, liver, &c. As regards the spinal cord, the blood-vessels of its dura mater were full of blood; those of its pia mater, especially the veins, distended; the veins of the cauda equina, varicose; the spinal cord in its whole extent, especially towards the cauda equina, covered with pus, and totally without vestige of arachnoid membrane; the substance of the cord itself reduced to thin pap.

The author appends to his narration of the above curious and important case some useful practical reflections. He points out the striking absence of objective symptoms of sufficient gravity or severity to account for either the alarming physical or psychical morbid phenomena. He suggests how extremely difficult it might have been, supposing the patient before or in the early stage of his malady to have received a slight fall, or a stroke on the head or spinal region, or had by accident taken or had administered to him any slightly deleterious substance, to determine before a legal tribunal to what cause or causes the subsequent death ought fairly to be ascribed; and it is easy to perceive, from a consideration of the case, that such an enquiry would, in the circumstances supposed, have been an exceedingly delicate one. The author recommends that in order to guide us in such emergencies, cases like the present should be most carefully investigated and kept in mind. In the above case the patient, although, according to official report, in good health up to the 17th and during the seven preceding years, was evidently in a condition of body predisposing in the highest degree to the most rapid and fatal disease. The rapid development of lesions in the other organs shows how speedily parts supplied by the peripheral nerves become disorganized when the central nervous masses themselves are attacked.

*Osterreich. Medicinische Wochenschrift*, No. IV. den 22 *Jänuar*, 1842.

---

*On the employment of the Sulphate of Alum in the treatment of some forms of Angina Pharyngea.* By M. Celestin Perrin.

It is by no means unusual for catarrhal affections, especially in damp situations, to leave behind them a sort of habitual chronic catarrh of the fauces. In these cases the mucous membrane is much injected, of a deep red, sometimes thickened, and the mucous follicles are very apparent and much developed. An adhesive mucus covers the parts and provokes a frequent and troublesome cough to effect its expectoration. The employment of alum gargles, of various strength, in these affections has for some years been often resorted to. M. Petrêquin, of the Hôtel Dieu, has practised the insufflation of four parts of alum to one of sugar with great success; and M. Perrin has used the same means with similar results.

Encouraged by the good effects of the application in chronic cases, M. Perrin has had recourse to it in those which are acute. He mixes equal parts of alum and sugar, and blows them through a quill against the back of the pharynx. It is always necessary that the point of the quill should be even with the uvula, since otherwise the sudden descent of the velum palati may close the passage and scatter the powder on the back of the tongue, where it excites nausea and efforts at vomiting. Even in cases where the febrile symptoms run very high, the difficulty of swallowing is extreme, and the patients have on former occasions been depleted and subjected to very severe treatment, this application a few times repeated has seemed to effect a cure, and a great amelioration of the symptoms has followed its employment even once. Two cases are related in illustration, and the writer concludes by asking whether equally favorable results might be expected from this practice in cases occurring in dry and hot countries, or whether there is something peculiar in the anginas of damp and rainy climates, as Lyons, which renders them peculiarly amenable to this mode of treatment.

*Bulletin Général de Thérapeutique. Mars*, 1842.

---

*On the Delirium following Acute Meningitis, and its Treatment by Cold Affusion.* By M. Recamier.

In the Hôtel Dieu, under the care of M. Recamier, was a man who was the subject of chronic delirium, unaccompanied by any sign of disease of the brain or its membranes. He had already had two attacks of a similar kind, the second more severe than the first, and the third than the second, but they all yielded to depletion, which in the last instance required to be repeated several times. In the same ward was another patient in whom delirium remained after symptoms of inflammation of the brain, and depended, in M. Recamier's opinion, on some modification in innervation produced by the disease, not on any continuance of the actual malady.

Two other similar cases are mentioned by him in which chronic delirium was removed by the employment of cold affusion. One was the case of a young lady in whom delirium existed unaccompanied by the signs of meningitis, but with irregular automatic movements of the hands, in which she crammed anything that was given her into her mouth. M. Recamier began the treatment with cold affusion of water at 68° Fahrenheit during five minutes, and gradually reduced the temperature to 50°, and prolonged the affusion for ten minutes. It was not, however, until affusion was practised at 39° that any effect was produced, but then the patient was seized with a tetanic spasm, and lost all consciousness. She was at once placed in bed, warmth was applied to the surface, and she speedily recovered, and asked for her mother, for whom she had before shown a marked aversion. Pen and paper being given her, she now wrote a perfectly sane letter, requesting her mother to visit her. The affusions were still continued at a temperature of about 64° for some days, and the patient recovered uninterruptedly.

In this case, which had followed an attack of meningitis, warm baths had been employed for a considerable time, without leading to any results. In the second case the same treatment was employed with success in a man who had become insane from spirit-drinking. In three different attacks the remedy had the effect of restoring his reason, but his intemperate habits were so deeply rooted that he eventually brought on a condition of permanent delirium, for which he was received into the Bicêtre.

*Gazette des Hôpitaux. Mars* 3, 1842.

---

*Extemporaneous Production of Milk.* By M. DICHOST.

M. DICHOST, a Russian chemist, proposed the following plan for the preservation and extemporaneous preparation of milk. He evaporates newly-drawn milk, at a very gentle heat, till it is all brought to a state of fine powder. It is then put into small glass bottles, which are completely filled and hermetically sealed, with ground glass stoppers. A small quantity of the powder thus obtained, dissolved in an appropriate quantity of water, affords on the instant a milk of very good quality. The powder will remain good for a great length of time.

*Gazette des Hôpitaux. Janvier* 22, 1842.

---

*On the External Application of Croton Oil.* By M. BOUCHARDAT.

WHENEVER it is required to use this method of counter-irritation, M. Bouchardat strongly recommends a plaster which has been much used by M. Chomel at the Hôtel Dieu, and which is thus prepared: Four parts of diachylon-plaster are melted at a very gentle heat, and while it is half liquid one part of croton oil is mixed with it, and the mixture is then spread in a thick layer on calico. Pieces cut from this may be applied to the skin, like ordinary sticking-plaster, and quickly produce an active irritation.

*Bulletin Général de Thérapeutique. Mars,* 1842.

---

*Tannin, as an Antidote for Strychnia.* By Dr. LÜDICKE.

THE great care requisite in the employment of strychnia is well known. Of this and of the benefits of tannin when an over-dose has been administered the following case is an illustration.

A woman, thirty-seven years of age, had complained for a considerable time of great tenderness and of a wandering pain in the course of the transverse and descending colon, for which opium and various other remedies were unsuccessfully employed. Dr. Lüdicke then ordered one twenty-fourth of a grain of nitrate of strychnia every hour. This dose, however, the patient ventured to increase, and continued to do so till she had taken half a grain of strychnia in the course of six hours. She was now suddenly seized with vertigo, and fell to the ground in a state of insensibility. A quarter of an hour afterwards she complained, though speaking with great difficulty, of having had a sensation as though her spine were being bent backwards, which had then passed off, leaving pain in the back, and tremor of the hands. The respiration was difficult, the pulse feeble and frequent, and the patient had a sensation of nausea with giddiness whenever she attempted to sit upright, and occasional vomiting. Ice was applied to the head, and half a grain of tannic acid was given every hour, at first in an effervescing mixture, on account of the sickness, afterwards in distilled water. After twelve grains had been taken the writer substituted for it a decoction of two ounces of oak bark in six ounces of water, with an ounce of syrup of cinnamon and a scruple of sulphuric æther. The symptoms of poisoning all disappeared, and with them the wandering pains from which the patient had previously suffered.

Mesner, in Dresden, recommends, as an antidote for strychnine, decoction of galls or of oak bark ; five ounces of which precipitate two grains of nitrate of strychnine. These decoctions likewise precipitate morphin, codein, brucin, veratrin, and many other vegetable alkaloids.

*Medicinische Zeitung. Märs* 16, 1842.

